# Maternal methionine supplementation during gestation alters alternative splicing and DNA methylation in bovine skeletal muscle

**DOI:** 10.1186/s12864-021-08065-4

**Published:** 2021-10-30

**Authors:** Lihe Liu, Rocío Amorín, Philipe Moriel, Nicolás DiLorenzo, Phillip A. Lancaster, Francisco Peñagaricano

**Affiliations:** 1 Department of Animal and Dairy Sciences, University of Wisconsin-Madison, 1675 Observatory Dr, Madison, WI 53706 USA; 2 University of Florida Genetics Institute, University of Florida, 32611 Gainesville, FL USA; 3 Range Cattle Research and Education Center, University of Florida, 33865 Ona, FL USA; 4 North Florida Research and Education Center, University of Florida, 32351 Marianna, FL USA; 5 Department of Clinical Sciences, Kansas State University, 66506 Manhattan, KS USA

**Keywords:** Differential isoform expression, Differential exon usage, Fetal programming

## Abstract

**Background:**

The evaluation of alternative splicing, including differential isoform expression and differential exon usage, can provide some insights on the transcriptional changes that occur in response to environmental perturbations. Maternal nutrition is considered a major intrauterine regulator of fetal developmental programming. The objective of this study was to assess potential changes in splicing events in the *longissimus dorsi* muscle of beef calves gestated under control or methionine-rich diets. RNA sequencing and whole-genome bisulfite sequencing were used to evaluate muscle transcriptome and methylome, respectively.

**Results:**

Alternative splicing patterns were significantly altered by maternal methionine supplementation. Most of the altered genes were directly implicated in muscle development, muscle physiology, ATP activities, RNA splicing and DNA methylation, among other functions. Interestingly, there was a significant association between DNA methylation and differential exon usage. Indeed, among the set of genes that showed differential exon usage, significant differences in methylation level were detected between significant and non-significant exons, and between contiguous and non-contiguous introns to significant exons.

**Conclusions:**

Overall, our findings provide evidence that a prenatal diet rich in methyl donors can significantly alter the offspring transcriptome, including changes in isoform expression and exon usage, and some of these changes are mediated by changes in DNA methylation.

**Supplementary Information:**

The online version contains supplementary material available at 10.1186/s12864-021-08065-4.

## Background

High-throughput mRNA sequencing technology offers a robust, efficient, and affordable way for detailed profiling of whole-transcriptome mRNA expression level [1]. The number of sequencing fragments that map to a given genomic element correlates directly with its abundance level. This type of quantification is commonly performed at the gene level, followed by the statistical detection of differentially expressed genes between conditions, which is well-known as differential expression analysis. However, this simple analysis may obscure the complex post-transcriptional regulatory dynamics in higher eukaryotes, where genes can produce multiple transcripts through alternative splicing to maintain functional complexity and protein diversity. Splicing patterns are constantly changing, enabling the organisms to respond to environmental perturbations [2, 3]. For instance, more than 90 % of human genes have been found having alternative splicing activities, and the isoform expression dynamics caused by alternative splicing has been associated with several diseases, including cancer [4]. Undoubtedly, the assessment of alternative splicing patterns can provide more detailed insights on transcriptional changes that occur in response to external perturbations.

The characterization of alternative splicing involves two types of measurement, namely differential isoform expression and differential exon usage. Differential isoform expression refers to changes in the absolute expression level of an isoform, while differential exon usage refers to changes in isoform proportions [5]. In both cases, the quantification of expression at a more precise level, such as isoform or exon instead of gene needs to be obtained. There are various methods and computational tools to study both isoform expression and exon usage [6–12]. Although some studies have compared different pipelines and/or workflows in either a descriptive way or an experimental way, no clear consensus has been made about the best workflow to use [5, 13–16]. Recently, Merino et al. [16] suggested that *DESeq2* and *DEXSeq* workflows could achieve high sensitivity for the analysis of isoform expression and exon usage, respectively.

Fetal programming refers to the fetal response to an intrauterine stimulus or insult, such as changes in nutrition, heat stress or exposure to disease, during a critical developmental period, which could induce permanent changes to the structure, physiology, and metabolism of the offspring [17, 18]. Maternal nutrition is recognized as a major intrauterine environmental factor and plays a critical role in fetal development, resulting in remarkable alterations of the developmental path [19]. The molecular mechanisms underlying fetal developmental programming due to maternal nutrition are not clear. However, there is growing evidence that maternal diets can modify the fetal epigenome, such as changing the DNA methylation, and these changes may lead to transgenerational phenotypic changes [20–22]. DNA methylation, the addition of a methyl group to the C-5 position of the cytosine (5^me^C), is a common epigenetic mark in many eukaryotes and occurs predominantly at CpG dinucleotides [19, 23]. DNA methylation in the gene promoter typically acts to repress gene expression, while DNA methylation in the gene body has been related to alternative splicing [24–26].

The main objective of this study was to evaluate whether a prenatal diet rich in methyl donors can affect alternative splicing patterns in the skeletal muscle of beef calves. Maternal diets consisted in a control diet, or a methionine-rich diet offered during the periconceptional and early gestation periods. Both muscle transcriptome and methylome were evaluated using next generation sequencing. We hypothesized that maternal methionine supplementation could alter the fetal epigenome, which in turn could induce significant changes in splicing events.

## Results

### RNA-sequencing analysis

About 50 million paired-end reads were generated from each *longissimus dorsi* muscle sample. Roughly 78 % of the reads were mapped to the ARSUCD1.2 bovine genome reference using the software Hisat2, and more than 71 % of the reads were mapped to the reference transcriptome created with the software RSEM (see Additional file 1).

### Differential isoform expression and enrichment analysis

**Fig. 1:**
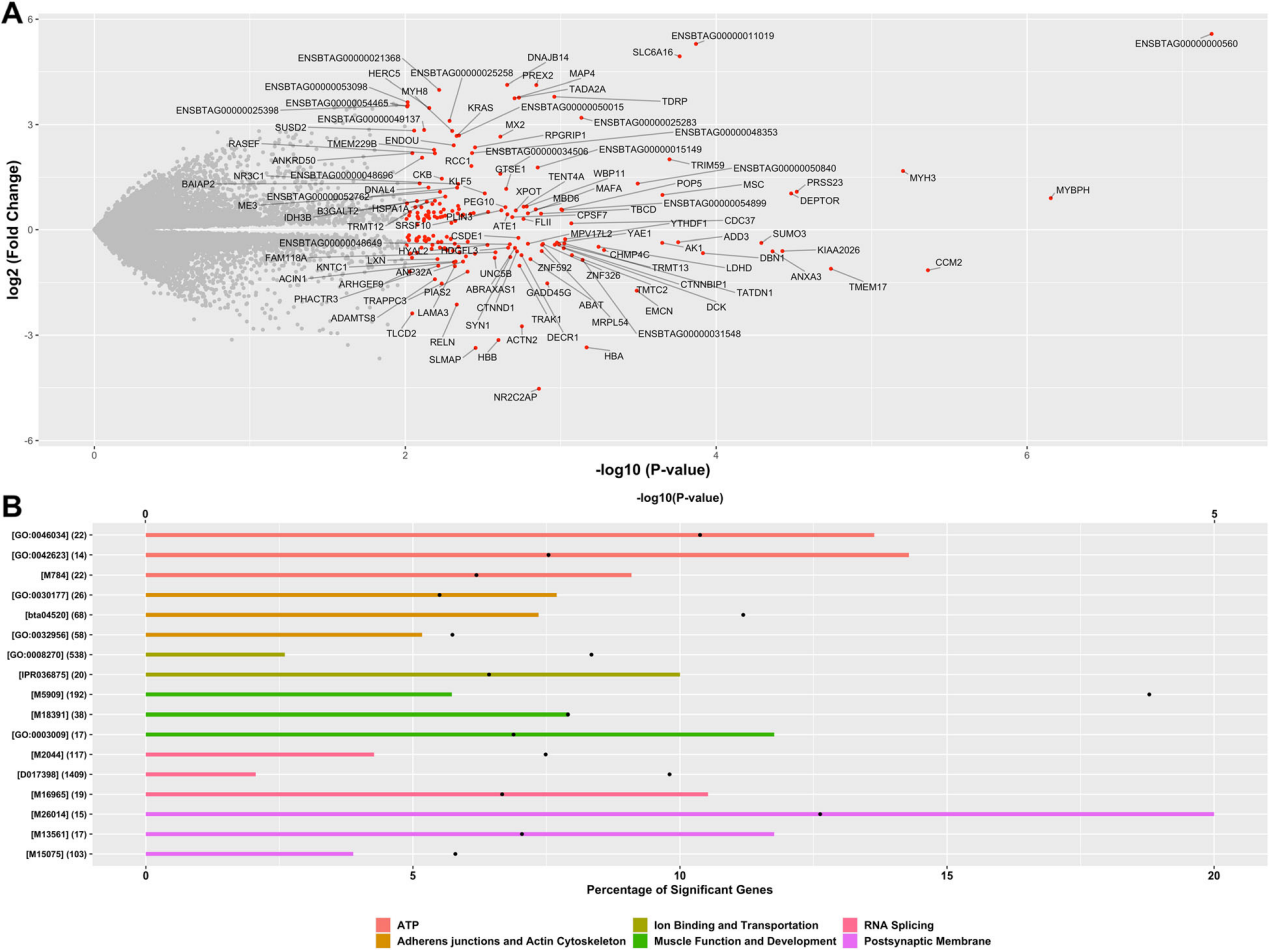
Differential isoform expression and functional characterization. **A** Volcano plot showing changes in isoform expression in bovine skeletal muscle exposed in utero to either a control diet (*n* = 9) or a methionine-rich diet (*n* = 10). The x-axis shows the magnitude of the change while the y-axis shows the statistical significance of the change. **B** Six gene annotation databases were analyzed, including Gene Ontology (GO), KEGG, Medical Subject Headings (MeSH), InterPro, Reactome and Molecular Signatures Database. The y-axis displays the term ID and the total number of genes in each functional term. The black dots represent the significance of enrichment (-log10 *P*-value, Fisher’s exact test, top x-axis) and the bars represent the percentage of significant genes in each functional term (bottom x-axis)

Only transcripts with 2 or more read counts in at least 9 biological replicates were considered. After removing lowly expressed isoforms, a total of 19,701 isoforms associated with 14,540 genes were evaluated for differential expression between maternal diets. As a result, 175 isoforms associated with 175 genes showed differential expression between maternal diets (*P*-value $\le$ 0.01, Fig. 1). Additional file 2 reports the full list of significant isoforms, including transcript gene ID, gene ID, gene name, log2-fold-change, and nominal *P*-value.

To gain more insight into the functional roles of the differentially expressed isoforms, and further characterize the biological processes that could be disturbed by maternal methionine supplementation, we performed a gene-set enrichment analysis. We evaluated the enrichment of functional terms with differentially expressed isoforms using six different databases, namely Gene Ontology (GO), Kyoto Encyclopedia of Genes and Genomes (KEGG), Reactome, InterPro, Medical Subject Headings (MeSH), and Molecular Signatures Database (MSigDB). The significant enrichment was analyzed using a Fisher’s exact test, a test of proportions based on the hypergeometric distribution. Figure 1 shows a list of gene-sets and functional categories that were significantly enriched with genes that showed differential isoform expression. We detected the enrichment of several functions and biological processes, including (i) muscle development, (ii) muscle physiology, (iii) ATP activities, (iv) RNA splicing, (v) postsynaptic membrane activities, (vi) adherens junctions and actin cytoskeleton, and (vii) ion binding and transportation. Additional file 3 shows the full list of significant functional terms, including term ID, term name, total number of genes, number of significant genes (i.e., genes with differentially expressed isoforms), percentage of significant genes, and Fisher’s *P*-value.

### Differential exon usage and enrichment analysis

**Fig. 2:**
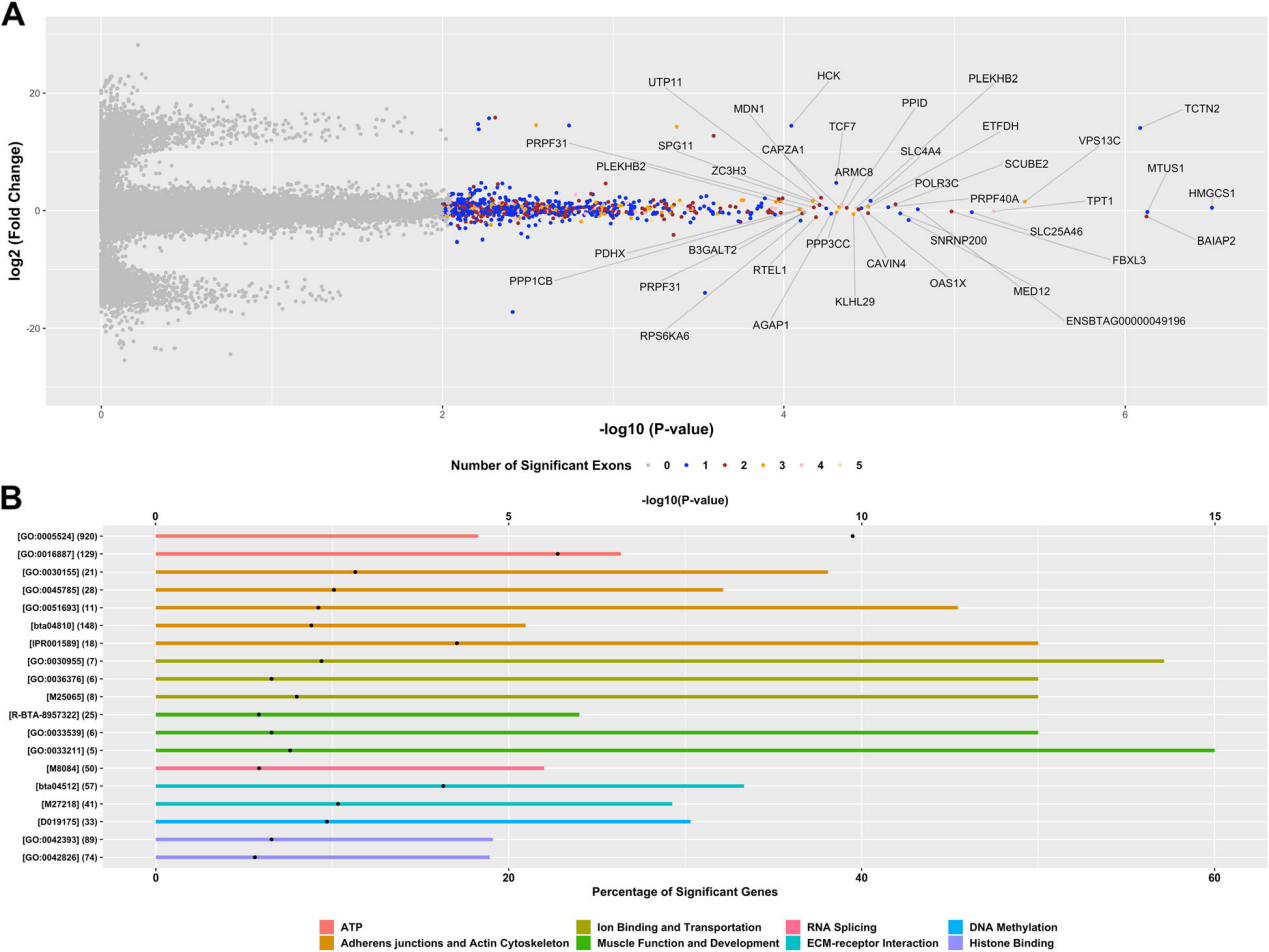
Differential exon usage and functional characterization. **A** Volcano plot showing changes in exon usage in bovine skeletal muscle exposed in utero to either a control diet (*n* = 9) or a methionine-rich diet (*n* = 10). The x-axis shows the magnitude of the change while the y-axis shows the statistical significance of the change. **B** Six gene annotation databases were analyzed, including Gene Ontology (GO), KEGG, Medical Subject Headings (MeSH), InterPro, Reactome and Molecular Signatures Database. The y-axis displays the term ID and the total number of genes in each functional term. The black dots represent the significance of enrichment (-log10 *P*-value, Fisher’s exact test, top x-axis) and the bars represent the percentage of significant genes in each functional term (bottom x-axis)

Only genes with 2 or more read counts in at least 9 biological replicates were considered. After removing lowly expressed genes, a total of 12,056 genes were evaluated for differential exon usage. As a result, a total of 1,745 exons associated with 1,290 genes showed differential usage between maternal diets (*P*-value $\le$ 0.01, Fig. 2). Additional file 4 shows the full list of significant exons, including the corresponding gene ID, gene name, log2-fold-change, and nominal *P*-value.

A gene-set enrichment analysis was performed using genes showing differential exon usage in order to reveal the biological mechanisms that could be impacted by maternal methionine supplementation. Notably, genes with differentially used exons were implicated in similar processes and functions that genes with differentially expressed isoforms, such as (i) muscle development, (ii) muscle physiology, (iii) ATP activities, (iv) adherens junctions and actin cytoskeleton, (v) ion binding and transportation, and (vi) RNA splicing. In addition, genes showing differential exon usage were also involved in DNA methylation, Histone binding, and ECM-receptor interaction. Figure 2 shows a subset of biological terms and functional categories that were significantly enriched with genes that showed differential exon usage. Additional file 5 shows the full list of significant terms, including term ID, term name, total number of genes, number of genes with differential exon usage, percentage of genes with differential exon usage, and Fisher’s *P*-value.

### DNA methylation analysis

Roughly 350 M paired-end reads per muscle sample were generated using whole-genome bisulfite sequencing. Reads were mapped to the latest bovine reference genome (ARS-UCD1.2) using the software *Bismark* yielding a 70 % mapping rate (see Additional file 1). A total of 5,136,556 cytosines in a CpG context were evaluated (read coverage $\ge$ 8), and 214,676 were identified as differentially methylated between maternal diets (*P*-value $\le$ 0.01). Following the ARS-UCD1.2 genome annotation, the cytosines under evaluation were categorized as (i) located within an exon, (ii) located within an intron, (iii) located within the promoter region (3 kb upstream the transcription start site), or (iv) located in an intergenic region. As a result, we targeted a total of 24,156 annotated genes that had at least one evaluated cytosine (either in exon, intron, or the promoter region), and 14,115 of these genes were found having at least one differentially methylated cytosine. Additional file 6 reports the full list of differentially methylated cytosines and the corresponding genomic regions. Additional file 7 reports the full list of transcripts evaluated in the analysis and the corresponding CpG count in exons and regulatory region. Additional file 8 reports the full list of exons evaluated in the analysis and the corresponding CpG count in exons, introns, and regulatory region.

### Alternative splicing and DNA methylation

**Fig. 3:**
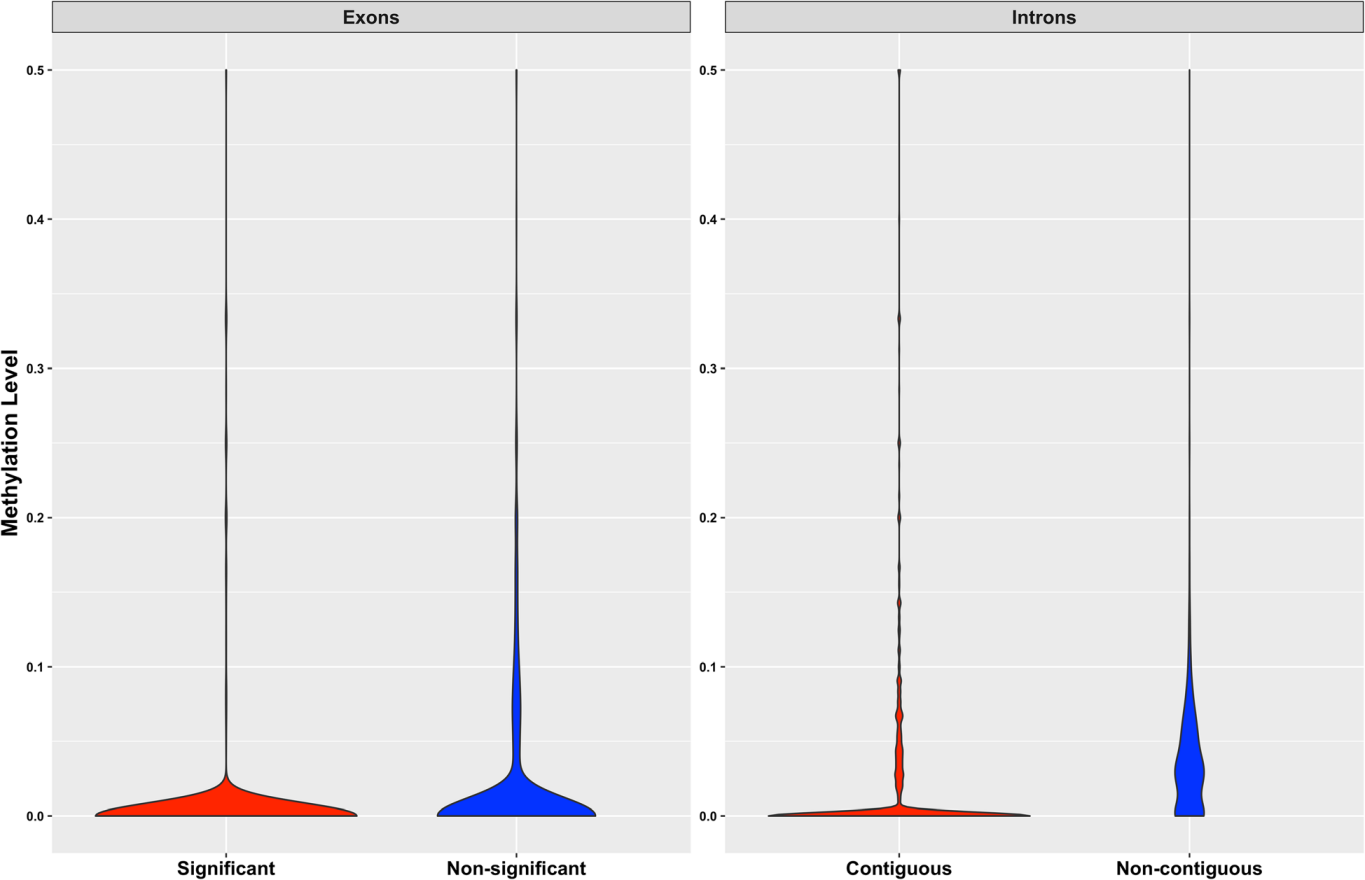
Association between DNA methylation and exon usage. Methylation level was calculated as differentially methylated cytosines divided by all the cytosines evaluated in a certain genomic region. Significant differences in methylation level were detected between significant and non-significant exons, and between contiguous and non-contiguous introns to significant exons (Kolmogorov-Smirnov test, *P*-value ≤ 0.001)

We evaluated the potential relationship between DNA methylation and alternative splicing, either as differential isoform expression or differential exon usage. For each gene under evaluation, we calculated the methylation level as the number of differentially methylated cytosines divided by the number of cytosines evaluated in each genomic region of interest, including exons, introns, or the promoter region. Notably, among the genes with at least one differentially used exon and methylation data (1,216 out of 1,290), methylation level in the significant exons was significantly different than the methylation level in the non-significant exons (Kolmogorov-Smirnov test, *P*-value $\le$ 0.001, Fig. 3). Similarly, methylation level in the introns that were contiguous to significant exons was significantly different than the methylation level in the non-contiguous introns (Kolmogorov-Smirnov test, *P*-value $\le$ 0.001, Fig. 3). On the other hand, among genes that had at least one differentially expressed isoform and had methylation data (125 out of 175), there were no differences in methylation levels among exons. Similarly, there were no differences in methylation level in the promoter region of the genes that showed differential isoform expression (*n* = 153) and those that did not show differential isoform expression (*n* = 12,569), nor between genes that showed differential exon usage (*n* = 1,185) and those that did not show differential exon usage (*n* = 9302).

**Fig. 4:**
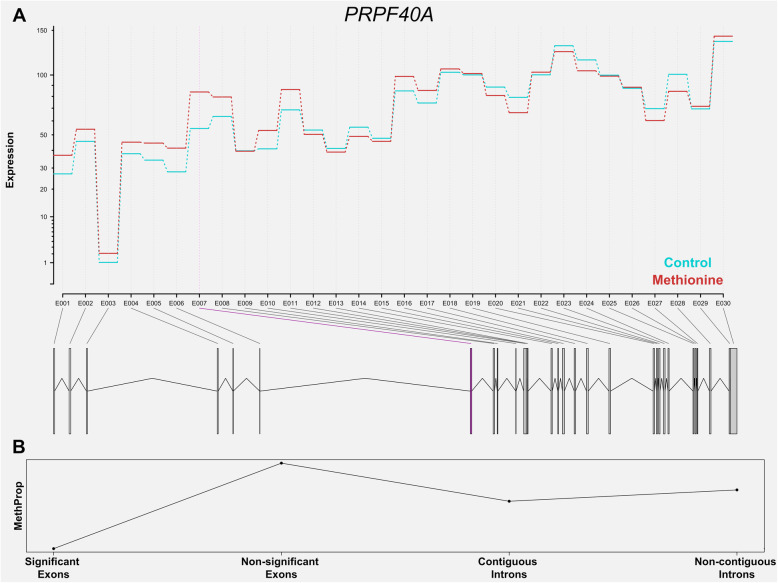
Illustration: Exon expression and DNA methylation in gene ***PRPF40A***. **A** Exon expression of gene *PRPF40A*. The x-axis displays the different exons (counting from 5’ to 3’) and the y-axis shows the corresponding expression estimates. **B** Methylation level of four genomic regions: significant exons, non-significant exons, contiguous introns, non-contiguous introns. Methylation level in a certain genomic region was calculated as differentially methylated cytosines divided by all the evaluated cytosines

To further visualize the relationship between DNA methylation and alternative splicing, we evaluated differential exon usage and differential DNA methylation at a single-gene level. As an illustration, Fig. 4 shows the exon expression level and DNA methylation status of *pre-mRNA-processing factor 40 homolog A* (*PRPF40A*), a gene that is highly involved in mRNA splicing and mRNA processing. Among the 30 exons and 26 introns annotated in the reference annotation, exon E007 was identified as differentially used between maternal diets. Notably, marked differences in methylation proportion were observed between this (significant) exon and the rest of the (non-significant) exons.

## Discussion

In high eukaryotes, alternative splicing is widely recognized for its pivotal roles in determining the relative ratio of isoform expression, and such dynamic post-transcriptional regulatory mechanism enables the host to generate sophisticated proteomes in response to environmental perturbations. In transcriptome studies, the evaluation of changes in splicing patterns can help to decode transcriptional mechanisms underlying complex traits from a unique perspective other than overall gene expression analysis. The present study was specially designed to detect changes in alternative splicing patterns due to maternal methionine supplementation. Maternal nutrition represents a major intrauterine environmental factor and altering maternal nutritional status during pregnancy can induce remarkable effects on fetal developmental programming. Here, we evaluated the changes in splicing events using two alternative approaches, namely differential isoform expression and differential exon usage. We functionally characterized the genes showing either differentially expressed isoforms or differentially used exons. We also investigated the link between DNA methylation and alternative splicing. Our results provide evidence that a prenatal diet rich in methyl donors can induce changes in alternative splicing patterns, and some of these changes are mediated by alterations in DNA methylation.

Maternal methionine supplementation significantly impacted alternative splicing patterns in the offspring’s muscle. Many significant genes are closely related to muscle development and muscle physiology, such as *TNNT3, PYGM, PFKM, ACTC1, CAPZA1, PALLD* and *MRAS*. For instance, gene *TNNT3* encodes the vertebrate fast skeletal troponin T protein, which is an important regulatory and structural component of thin filaments in skeletal muscle [27]. Genes *PYGM* and *PFKM* play essential roles in glycolytic metabolism, which serves as the primary source of energy in the muscle. Gene *PYGM* encodes the muscle-specific isoform of glycogen phosphorylase, which produces α D-Glucose 1P from glycogen [28]. Gene *PFKM* encodes the phosphofructokinase, which controls the rate‐limiting step in glycolysis, the conversion of fructose‐6‐phosphate to fructose‐1,6‐diphosphate [29]. Gene *ACTC1* is the predominant striated α-actin isoform in the heart but is also expressed in developing skeletal muscle, where α-actin is a major constituent of the contractile apparatus [30]. Gene *CAPZA1* encodes for the alpha subunit of the barbed-end actin binding protein; it regulates growth of the actin filament by capping the barbed end of growing actin filaments [31]. Gene *PALLD* encodes a cytoskeletal protein that is required for organizing the actin cytoskeleton and could affect the number and size of actin bundles, as well as muscle cell proliferation and differentiation [32]. Gene *MRAS* encodes a member of the Ras family of small GTPases, these membrane-associated proteins function as signal transducers in multiple processes, including cell growth and differentiation. Note that *MRAS* is a member of the GO term *muscle organ development* (GO: 0007517). Overall, these results indicate that maternal methionine supplementation altered the alternative splicing patterns of many genes that are closely related to muscle development and muscle physiology.

Interestingly, the functional characterization analysis indicated that some of the genes showing either differential isoform expression or differential exon usage are directly implicated in muscle development. For example, the functional term *genes involved in development of skeletal muscle/myogenesis* (MSigDB: 5909) was significantly enriched with genes impacted by maternal methionine supplementation. There is growing evidence that myogenesis is one of the most important mechanisms by which maternal nutrition can impact muscle developmental programming. Fahey et al. [33] has reported that maternal undernutrition could result in fewer myosin heavy-chain fast fibers (MHC-fast) and significantly more myosin heavy-chain slow fibers (MHC-slow) in both *lumbar multifidus* and *vastus lateralis* muscles in lambs. Another sheep study showed that fetuses from ewes receiving 50 % of total maternal digestible nutrients from d 28 to 78 of gestation had lower numbers of myofibers compared to controls [34]. Another relevant functional term impacted by methionine supplementation was *metabolism of lipids* (MSigDB: 27,451), and this provides further evidence that prenatal diets can modulate lipid content in the offspring muscle. For example, studies in sheep have reported that maternal obesity during gestation can induce an increased lipid content in offspring muscle at 22 mo. of age [35, 36], whereas a nutrient restriction from d 28 to 78 of gestation increased the intramuscular triglyceride concentration of the longissimus muscle at 8 mo. of age [37]. Note that myogenesis and intramuscular lipid deposition directly impact lean muscle mass and marbling, and hence, any perturbations in these physiological processes due to maternal nutrition may have long-term consequences, impacting muscle growth and meat quality.

Several functional terms identified as significant are closely related to key muscle cell functions and processes, including ATP activities, ion binding and transportation, adherens junctions and actin cytoskeleton, extracellular matrix receptors, postsynaptic membrane, and histone binding. Previous studies have shown that these cell activities may be impacted by maternal nutrition. For example, Lillycrip et al. [38] reported over-representations of genes related to cation transmembrane transporter activity, anion transmembrane transporter activity and ATPase activities among the differentially expressed genes identified in response to maternal protein restriction diet in rats. Also, Max et al. [39] identified actin cytoskeleton related pathways associated with differentially expressed genes in newborn rats as response to maternal vitamin D deficiency, such as *regulation of actin cytoskeleton organization* and *stimulation of cytoskeleton organization.* In addition, Glendining et al. [40] reported that a high-fat diet in mice altered histone binding at the promoter region of gene *Oxtr* in the offspring hippocampus.

More than 4 % of all evaluated cytosines were found to be differentially methylated due to a maternal methionine-rich diet. Interestingly, the functional characterization of genes with differential exon usage showed that the gene-set *DNA methylation* (MeSH: D019175) was significantly impacted. Indeed, some of the significant genes are directly implicated in DNA methylation, such as *PP2Ac* and *RTEL1*. For example, Sunahori et al. [41] showed that gene *PP2Ac* regulates the expression of *DNMT1*, one of the three major methyltransferases implicated in DNA methylation. Similarly, a recent study showed that gene *RTEL1* alters the abundance and localization of telomeric repeat containing RNAs, affecting in this way the formation of chromatin loops, which in turn prevent the methylation of gene promoters [42]. It is well-documented that maternal nutrition can alter epigenetic marks of the fetal genome, such as DNA methylation [20], and our findings provide additional evidence. Notably, we revealed a significant association between DNA methylation and differential exon usage. Indeed, significant differences in methylation level were found between significant and non-significant exons. Similarly, significant differences were found between contiguous and non-contiguous introns to significant exons. There is growing evidence that DNA methylation is associated with alternative splicing. For instance, Maunakea et al. [26] have shown that DNA methylation is significantly enriched in included exons compared to excluded exons during alternative splicing. These authors suggest that DNA methylation affects exon inclusion by recruiting MeCP2 and subsequent HDAC activities. In addition, Shukla et al. [25] have proposed a mechanism where a DNA-binding protein, namely CCCTC-binding factor, can promote inclusion of exons by mediating local RNA polymerase II pausing for alternative splicing. Of special interest, Lev Maor et al. [43] have proposed another mechanism of how DNA methylation can influence alternative splicing which involves the formation of a protein bridge by heterochromatin protein 1 that recruits splicing factors onto transcribed alternative exons. Interestingly, our functional characterization of significant genes reported several gene-sets related to RNA splicing, including *RNA splicing* (MSigDB: 8084), *spliceosome* (MSigDB: 2044) and *spliceosomal complex* (MSigDB: 17,478 and MSigDB: 15,787). Similarly, some of the most significant genes are directly implicated in RNA splicing, such as *SRSF10*, *CHERP* and *DHX8*. Gene *SRSF10* encodes a serine/arginine-rich splicing factor that promotes exon skipping during the pre-mRNA alternative splicing process [44]. Gene *CHERP* encodes an endoplasmic reticulum protein that is involved in RNA processing, including the processing of capped intron-containing pre-mRNAs [45–47]. Gene *DHX8* encodes an ATP-dependent RNA helicase that plays an important role in the alternative splicing process, it localizes at the periphery of the spliceosomes and is involved in the release of mature mRNAs prior to their export from the nucleus [48, 49]. Overall, our findings provide evidence that maternal methionine supplementation can induce changes in the offspring epigenome, including DNA methylation marks, which in turn modulate alternative splicing events.

## Conclusions

Our study demonstrated that a periconceptional and early gestational diet rich in methyl donors can significantly alter splicing patterns in skeletal muscle of the progeny. The functional characterization of the disturbed genes revealed that several biological terms related to muscle development and muscle physiology were significantly impacted, including myogenesis, ATP activities, and ion binding and transportation. Additionally, some of the impacted genes were implicated in DNA methylation and RNA splicing activities. Interestingly, some of the changes in alternative splicing patterns were associated with changes in DNA methylation. To the best of our knowledge, this is the first study that investigates the link between maternal nutrition, DNA methylation and alternative splicing. Our findings provide evidence that alternative splicing patterns can be disturbed by maternal nutrition, and some of these adaptations are mediated by alterations in the DNA methylation.

## Methods

### Ethics statement

 All the animal procedures used in this study were approved by the Institutional Animal Care and Use Committee (IACUC #2,014,408,583) of the University of Florida. All experiments were performed in accordance with relevant guidelines and regulations.

### Animals, experimental design, and maternal diets

 The feeding trial was performed at the University of Florida (UF) Range Cattle Research and Education Center (Ona, Florida, USA). Brangus-Angus cows from UF/Ona were fed one of two diets from days − 30 to + 90 relative to the beginning of the breeding season. The diets consisted of (i) a control diet based on limpograss hay (*Hemarthria altissima*) with the supplementation of molasses and urea (22 % crude protein, 1.7 kg per head per day), or (ii) a methionine-rich diet equal to the control diet but supplemented with 10 g per head per day of MetaSmart Liquid (Adisseo, Alpharetta, GA) providing 3.7 g per head per day of rumen-protected methionine [50]. Longissimus dorsi muscle samples were collected from a total of 20 bull calves, 10 animals per maternal diet, at 1 month of age. Approximately 50 mg of muscle samples were collected from the longissimus dorsi muscle located above the 11th and 12th rib using a Tru-Cut biopsy device. Immediately after sample collection, muscle samples were snap-frozen with liquid nitrogen. Muscle samples were stored at -80˚C until RNA extraction. After sampling, the bull calves were released.

### RNA extraction, library preparation and sequencing

Total RNA was successfully extracted, processed, and sequenced from a total of 19 bull calves, 9 from the maternal control diet and 10 from the maternal methionine-rich diet. Qiagen RNeasy Mini kit was used for total RNA extraction and quality of the yielded RNA was evaluated using Agilent 2100 Bioanalyzer (Agilent Technologies, Inc.) [22]. RNA-sequencing libraries were prepared from 50 ng RNA samples using a poly(A) capture method and then sequenced using Illumina’s HiSeq 3000 at the University of Florida. The RNA-sequencing data can be accessed by NCBI GEO with the accession number GSE116974.

### RNA-seq quality control and editing

The software FastQC (v0.11.7, Babraham Bioinformatics, UK) was used to evaluate the quality of the sequencing reads, and the software Trim Galore (version 0.4.4, Babraham Bioinformatics, UK) was used for adaptor removal and trimming using the following parameters: −−paired, −−clip_ R1 10, −−clip_R2 10, −−three_prime_clip_R1 10, −−three_ prime_clip_R2 10, and −−length 20.

### Isoform expression: mapping, quantification, and statistical analysis

 Isoform expression was analyzed using RSEM and DESeq2. First, the method *rsem-prepare-reference* [51] was used to extract reference transcripts from the latest bovine reference genome (ARS-UCD1.2) annotation. Then, the method *rsem-calculate-expression* was used to quantify isoform expression. The *R* package DESeq2 (v1.30.0) [10] was used to identify differentially expressed isoforms between maternal diets using the following steps: (i) estimation of normalization factors (median of ratios method), (ii) estimation of dispersion parameters, (iii) fit of negative binomial generalized linear models, and finally (iv) use of Wald tests to detect differentially expressed isoforms.

### Exon usage: mapping, quantification, and statistical analysis

Exon usage was analyzed using Hisat2 and DEXsEq. Sequencing reads were mapped to the ARS-UCD1.2 bovine reference genome using the software Hisat2 (v2.1.0) [52]. Then, the python script *dexseq_count.py* implemented in the *R* package DEXseq (v1.36.0) [9] was executed to quantify exon expression. The *R* package DEXSeq was also used to evaluate differential exon usage, following these steps: (i) estimation of normalization factors (median of ratios method), (ii) estimation of dispersion parameters, (iii) fit of negative binomial generalized linear models, and finally (iv) use of likelihood ratio tests to detect differentially used exons between maternal diets.

### Gene-set enrichment analysis

Functional terms from various biological databases, including Gene Ontology (GO) [53], Kyoto Encyclopedia of Genes and Genomes (KEGG) [54], InterPro [55], Reactome [56], Medical Subject Headings (MeSH) [57] and Molecular Signatures Database (MSigDB) [58], were examined using a Fisher’s exact test, a test of proportions based on the cumulative hypergeometric distribution. Genes showing differential exon usage (*P*-values $\le$ 0.01) or differential isoform expression (*P*-values $\le$ 0.01) were tested against the background set of all expressed genes. All these analyses were performed using the *R* package EnrichKit (https://github.com/liulihe954/EnrichKit).

### DNA extraction, library preparation and sequencing

Total DNA was successfully extracted, processed, and sequenced from a total of 16 bull calves, 7 from the maternal control diet and 9 from the maternal methionine-rich diet. DNA extraction, library construction, bisulfite treatment and sequencing were performed by Novogene Bioinformatics Technology Co., Ltd. (Beijing, China) [22]. Libraries were sequenced with Illumina’s HiSeq 3000 using 150-bp paired-end reads. Whole-genome bisulfite sequencing data can be accessed by NCBI GEO with the accession number GSE117194.

### Whole-genome bisulfite-seq quality control and mapping

The software FastQC (v0.11.7, Babraham Bioinformatics, UK) was used for the quality control of the sequencing reads and the software Trim Galore (v0.4.4, Babraham Bioinformatics, UK) was used for adaptor removal and trimming when needed. After editing, the resulting paired-end sequencing reads were aligned to ARS-UCD1.2 bovine reference genome using the software Bismark (v0.17.0, Babraham Bioinformatics, UK) [59]. Duplicated read alignments were detected and removed using the Bismark tool *deduplicate_bismark*. The Bismark tool *methylation extractor* was used for methylation calling using the following parameters: --paired-end, --comprehensive, --bedGraph, and --cytosine_report.

### Differentially methylated cytosines, exons and isoforms

Differentially methylated cytosines between maternal diets were identified using a logistic regression implemented in the *R* package Methylkit (v1.0.0) [60]. Only cytosines with read coverage $\ge$ 8 in a CpG context were evaluated. Differentially methylated cytosines were defined as those having *P*-value ≤ 0.01. Both significant and non-significant cytosines were mapped to different genomic features, including exons, introns, and promoter regions (3 kb upstream to the transcription start site), using the *R* package rtracklayer (v1.50.0) [61]. We then calculated the methylation level of a given genomic feature as the ratio of differentially methylated cytosines to all the cytosines evaluated in that specific region. For genes with at least one differentially used exon, we calculated the methylation level for: (i) the exons that were differentially used, (ii) the exons that were not differentially used, (iii) the introns that were contiguous to differentially used exons, and (iv) the introns that were non-contiguous to differentially used exons. For genes with at least one differentially expressed isoform, we calculated the methylation level for: (a) exons included in the differentially expressed isoform, and (b) exons not included in the differentially expressed isoform. We also evaluated the methylation level in the promoter region of genes that showed either differential exon usage or differential isoform expression.

## Supplementary Information

**Additional file 1.** Mapping Stats.xlsx: Summary of both RNA sequencing and whole-genome bisulfite sequencing.

**Additional file 2.** Significant Genes DIE.xlsx: Summary of significant genes detected in the differential isoform expression analysis.

**Additional file 3.** Enrichment DIE.xlsx: List of significant terms detected in the functional characterization of genes that showed differential isoform expression.

**Additional file 4.** Significant Genes DEU.xlsx: Summary of significant genes detected in the differential exon usage analysis.

**Additional file 5.** Enrichment DEU.xlsx: List of significant terms detected in the functional characterization of genes that showed differential exon usage.

**Additional file 6.** Differentially Methylated Cytosines.xlsx: List of differentially methylated cytosines.

**Additional file 7.** DIE and Methylation level.xlsx: List of CpG sites classified by genomic regions and grouped by isoforms.

**Additional file 8.** DEU and Methylation level.xlsx: List of CpG sites classified by genomic regions and grouped by exons/introns.

## Data Availability

Bisulfite-Seq (GSE117194) and RNA-Seq (GSE116974) data are available on NCBI GEO with accession number (SuperSeries) GSE117195.

## Abbreviations

DIE: Differential isoform expression
DEU: Differential exon usage
